# Characterization of Trimmed Nerve Morphology Using High-Resolution Imaging: Comparison of Three Surgical Instruments

**DOI:** 10.1016/j.jhsg.2026.101003

**Published:** 2026-04-03

**Authors:** Rasa Zhukauskas, Brandon S. Smetana, Adam B. Strohl, Sunishka M. Wimalawansa, Eitan Melamed, Amy M. Moore, Fraser J. Leversedge, Youssra Marjoua, Bauback Safa

**Affiliations:** ∗ Axogen Corporation, Alachua, FL; † Indiana Hand to Shoulder Center, Indianapolis, IN; ‡ Philadelphia Hand to Shoulder Center, Philadelphia, PA; § Wright State University, Boonshoft School of Medicine/Miami Valley Hospital Department of Orthopaedics, Division of Plastic Surgery, Dayton, OH; ‖ NYC Health + Hospitals Elmhurst, Queens, NY; ¶ Department of Plastic and Reconstructive Surgery, The Ohio State University College of Medicine, Columbus, OH; # Department of Orthopedic Surgery, University of Colorado School of Medicine, Aurora, CO; ∗∗ Department of Orthopaedic Surgery, The Cleveland Clinic, Cleveland, OH; †† The Buncke Clinic, San Francisco, CA

**Keywords:** Nerve cutting, Nerve repair, Nerve trimming, Nerve zone of injury, Nerve reconstruction

## Abstract

**Purpose:**

Trimmed proximal and distal nerve stumps should be morphometrically matching and show evidence of healthy fascicular tissue to potentiate nerve regeneration. We hypothesized that differences exist between nerve trimming instruments, providing guidance for optimal nerve-trimming techniques.

**Methods:**

Three common surgical instruments were used to trim nerves in three human upper-extremity specimens: a slotted neurotome, a no. 11 surgical blade with a tongue depressor, and super-cut serrated microscissors. Peripheral nerves in flexor tendon zones II and V of the hand were trimmed by surgeons experienced in microsurgery. Microcomputed tomography was used to evaluate the length of damage of the retained nerve stump of trimmed nerves. This damage was measured as the distance from the end of the trimmed nerve to the cross-sectional level with circumferentially intact epineurium and recovered fascicular morphology, which was compared between instruments. The transverse cut end of the nerve was also qualitatively examined using cryoscanning electron microscopy for fascicular distortion and surface roughness.

**Results:**

The length of intraneural damage of the proximal and distal nerve stumps was similar between cutting instruments in zone II and in pooled samples (zone II and zone V). In zone V nerve samples, length of intraneural damage was significantly more variable in the scalpel-trimmed samples compared to the neurotome-trimmed and scissor-trimmed samples. Additionally, the cryoscanning electron microscopy images showed that the cut end of nerves trimmed with a scalpel or neurotome had less apparent fascicular distortion and roughness than nerves trimmed with super-cut serrated microscissors.

**Conclusions:**

Neurotome-trimmed nerves were the only group that exhibited transverse nerve end surface consistency, smoothness, and less fascicular distortion. We found that use of a surgical blade improves the quality of nerve preparation for repair or reconstruction over the use of super-cut serrated microscissors.

**Type of study/level of evidence:**

Basic Science V.

Poor-quality nerve coaptations may cause suboptimal matching between the proximal and distal nerve ends required for appropriate axonal regeneration across a nerve gap, potentially causing development of a neuroma-in-continuity. Preparation of nerve ends for surgical repair or reconstruction should be completed with instrument(s) that minimize damage to the nerve endings and remove damaged neural tissue until pouching fascicles are visible.^1^, ^2^, ^3^ Adequate preparation of nerve ends for reconstruction or repair are required to prevent scar formation, as scar may reduce axonal outgrowth and the potential for successful nerve regeneration.^3^

There are limited studies that scientifically compare the methods and instruments used to harvest nerve grafts or to trim damaged nerve ends in preparation for nerve repair or reconstruction.^4^ Surgical instruments commonly used to perform nerve end resection for nerve harvest or excision predominately include scalpel blades, in addition to microsurgical or tenotomy scissors, neurotomes, and razor blades.^5^ Improved retention of normal fascicular and epineural integrity may be obtained with neurotmesis using a razor blade over scissors.^4^ Effective neuroma prevention was noted when nerves were trimmed with lasers or scissors versus using electrocoagulation or cryoneurolysis.^6^ Previously, high-definition photomicrography with 50× magnification evaluated five surgical instruments (no. 15 blade scalpel with a tongue depressor, microvascular scissors, tenotomy scissors, iris scissors, and an Accurate Surgical & Scientific Instruments [ASSI] slotted, circumferential neurotome) and found a higher rate of acceptable cuts produced from the ASSI neurotome.^1^

Ultimately, the goal of preparing nerve ends for reconstruction or repair should focus on reproducibly creating even surfaces with limited fascicular distortion and intraneural damage, where the nerve faces match and fascicles align.^3^ Rose et al^3^ found that smoother surfaces of the trimmed nerve ends showed less fascicles protruding beyond the epineural ends by quantifying the surface texture. It is a common practice for surgeons to resect the injured nerve ends until punctate bleeding and pooching fascicles are visible.^3^ A previous study suggests conventional methods of nerve trimming by stabilizing the nerve stump with forceps and trimming back with a no. 11 blade or microscissors should be investigated critically.^7^ However, visual assessments of the prepared nerve face often rely on evaluation methods that are inherently limited, such as visual evaluation via loupe or surgical scope magnification. We hypothesized that fascicular integrity at the surface of the trimmed nerve end and the length of intraneural damage would be different between cutting methods and instruments. To visualize and to quantify this damage, we used microcomputed tomography (micro-CT) to measure intraneural damage depth and cryoscanning electron microscopy (cSEM) to qualitatively assess fascicular distortion and surface roughness.

## Materials and Methods

**Figure 1:**
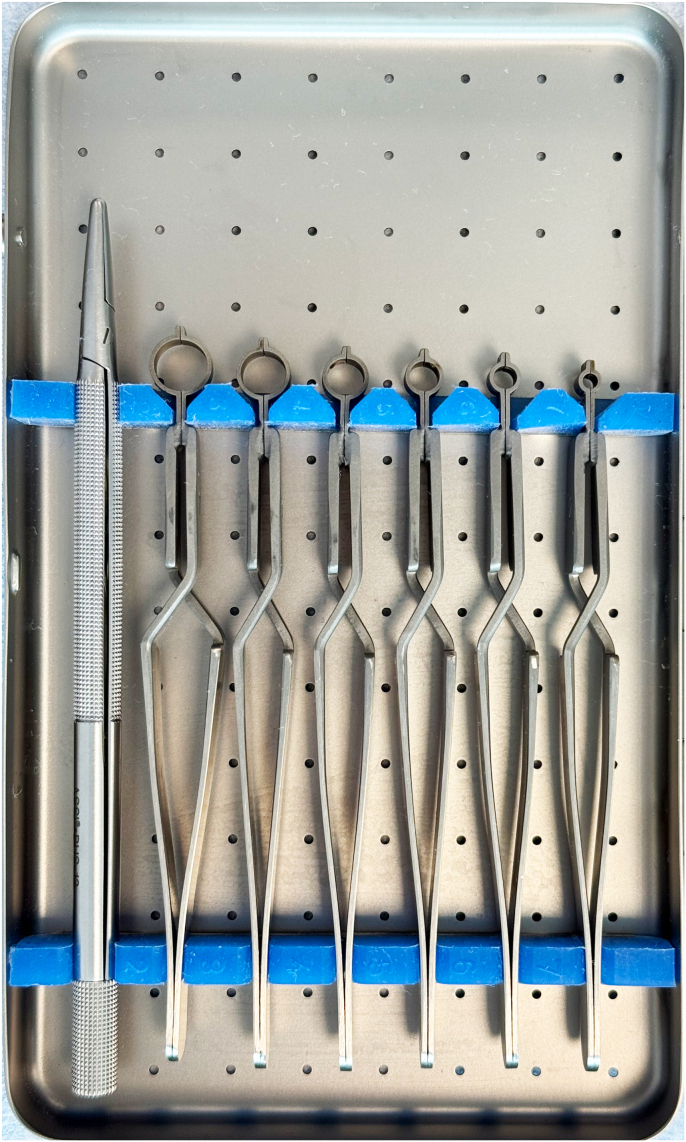
ASSI neurotome. To use the neurotome, the nerve is stabilized with a diameter matched-nerve holding forceps. An ASSI disposable blade is inserted and secured in a blade holder. The blade then is passed through a blade guiding slot within the nerve holding forceps using a sawing motion to transect/trim the nerve.

Three upper-extremity specimens were used in the evaluation of nerve-trimming instruments in flexor tendon anatomical zones II and V.^8^ These fresh frozen cadaveric specimens were from male donors between 51 and 61 years old with a body mass index between 21.5 and 26.4 consented for research. Nerve samples for analysis were exposed and trimmed with the predetermined instruments under loupe magnification by a group of seven surgeons experienced in peripheral nerve repair. Trimming instruments included ASSI neurotome (Fig. 1, catalog no. ASSI.NHS1, Accurate Surgical & Scientific Instruments Corp., Westbury NY), no. 11 scalpel blade with the nerve stabilized with forceps on a tongue depressor and cut using a guillotine technique, and super-cut serrated microscissors with the nerve held by forceps. All nerve cutting techniques were performed perpendicular to the nerve axis.

Both ends of each nerve segment (approximately 2 cm long) were trimmed from intact nerves (Table 1) with one of the selected instruments (scalpel, scissors, or neurotome). Trimmed nerve segments were stored in 10% neutral buffered formalin. Before imaging, samples were rinsed in 1× PBS and exposed to 1.5% buffered Lugol (Heiltropfen, pharma grade, catalog no. 12298-68-9) for 9–10 days.^9^ Micro-CT imaging was performed using SkyScan 1272 CMOS edition scanning system at Micro Photonics (Allentown, PA) using previously developed system settings.

**Table 1:** Nerve-Trimming Instrument and Sample Distribution

Trimming Instrument (N Segments, n samples)	Zone (N Segments, n samples)	Nerve (N Segments, n samples)	Specimen ID
Neurotome (N = 4; n = 8)	Zone II (N = 2; n = 4)	Digital II (N = 1; n=2)	Specimen 3
		Digital II (N = 1; n=2)	Specimen 3
	Zone V (N = 2; n = 4)	Median (N = 1; n = 2)	Specimen 1
		Ulnar (N = 1; n = 2)	Specimen 1
Scalpel (N = 4; n = 8)	Zone II (N = 2; n=4)	Digital I (N = 1; n = 2)	Specimen 3
		Digital IV (N = 1; n = 2)	Specimen 3
	Zone V (N = 2; n = 4)	Median (N = 1; n = 2)	Specimen 1
		Ulnar (N = 1; n = 2)	Specimen 1
Scissors (N = 4; n = 8)	Zone II (N = 2; n = 4)	Digital I (N = 1; n = 2)	Specimen 3
		Digital IV (N = 1; n = 2)	Specimen 3
	Zone V (N = 2; n = 4)	Median (N = 1; n = 2)	Specimen 2
		Median (N = 1; n = 2)	Specimen 2

N, number of nerve segments; n, number of trimmed nerve ends used for micro-CT examination (samples).

**Figure 2:**
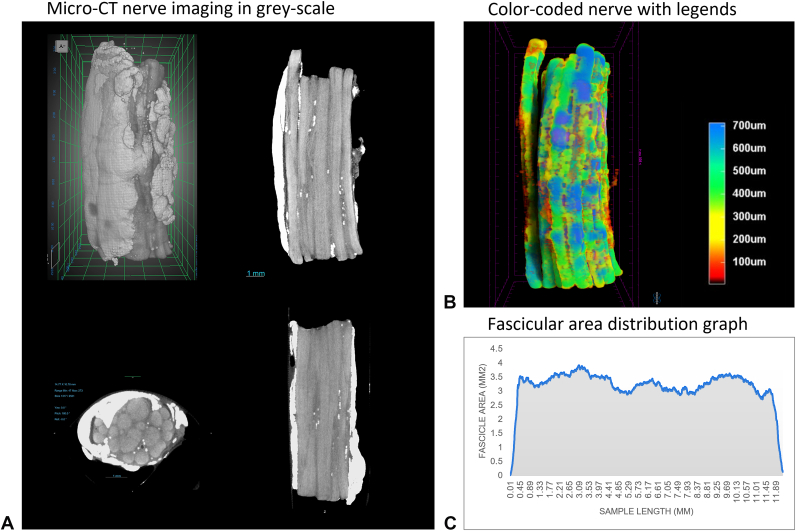
**A** Micro-CT gray-scale nerve images. **B** Color-coded nerve and color code legend indicating of fascicle size in μm. **C** Output of fascicular area distribution, where low radiodensity is indicated by a decreased area.

During micro-CT imaging, each nerve segment was fixed to a carousel base located between the X-ray source and the detector, which was rotated 360° around its axis to capture two-dimensional projection images. The radiodensity of fascicular structures was enhanced using an iodine-based contrast agent that has an affinity to lipid-containing anatomical structures such as adipose tissue and myelin.^9^ An automated segmentation based on gray-scale values representing fascicles was performed with Bruker CTvox 3-dimensional Suite software (Bruker, Ettlingen, Germany) to identify fascicular structures. Adipose tissue and other nonfascicular tissues were removed from the images. Nerve fascicle thickness measurements were recorded 5 μm apart and output into two-dimensional color-coded images. Images were reconstructed to create three-dimensional renderings and videos for each sample (Fig. 2). Radiographs were analyzed using Dragonfly three-dimensional World software (v 2024.1, Comet technologies, Canada).

Trimmed nerves were examined for tissue intraneural damage introduced by the surgical instruments. Each end of the nerve segment was examined as an independent sample, ie, two samples were imaged per nerve segment. Intraneural damage was evaluated and measured using high-resolution tomograms with Dragonfly three-dimensional software. Intraneural tissue damage was defined as the distance from the end of the trimmed nerve to the cross-sectional level with circumferentially intact epineurium and recovered fascicular radiodensity. Fascicular structure recovery to normal nerve appearance was tracked in cross-sectional planes. Areas of damaged epineurium were identified by decreases in radiopacity until full recovery.^10^ Damage was confirmed by comparing software-generated fascicular area distribution within cross-section segmentation images of each sample and visual interpretation of radiographic changes present in the three-dimensional renderings of each sample when compared to the central portion of the nerve segment (“normal nerve” area), which was verified by a qualified radiologist.

To perform cSEM, the ethane frozen samples were plunged into the PrepDek workstation liquid nitrogen slush at –210 °C under vacuum and immediately transferred to the cryopreparation chamber (PP3010, Quorum Technologies). The frozen tissue was sublimated at –60 °C for 15 minutes and sputter coated with platinum for 60 seconds at 10 mA current in an argon atmosphere. The cryopreparation chamber returned to –145 °C at a vacuum of >10 mbar, after which the sample was transferred to the nitrogen gas-cooled cold stage inside the SEM chamber at –140 °C (SU5000, Hitachi High Technologies, America). The sample remained frozen at –140 °C under high vacuum conditions and 5 keV accelerated voltage during the imaging. cSEM images of the cut nerve end were evaluated qualitatively for fascicular distortion and surface roughness.

A power analysis was run based on the average intraneural damage between groups in a pilot study. The target power of the study was 0.8, which required n = 4 per group and per zone of injury. Selected continuous data were expressed as group mean ± SD and 95% confidence interval. A one-way analysis of variance test was used to evaluated differences between groups, with a Tukey correction for multiple comparisons. A power evaluation after study data collection showed an achieved power of 0.869. A *P* value < .05 was considered statistically significant.

## Results

**Figure 3:**
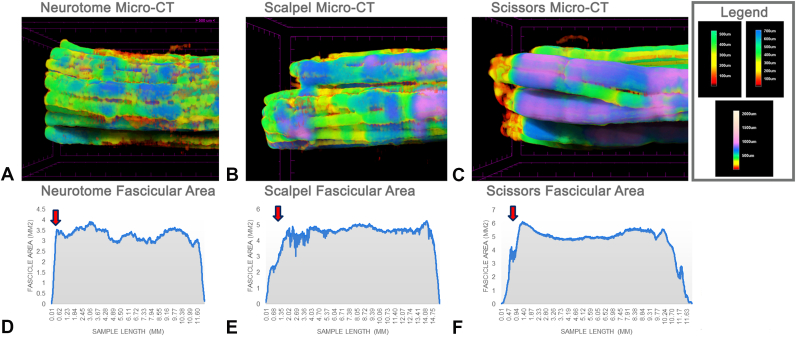
Micro-CT images and nerve cross-sectional area recovery curves of the nerves from flexor zone V trimmed with three surgical instruments. Micro-CT imaging of nerves trimmed with the following: **A** neurotome, **B** scalpel, and **C** scissors. **D** Nerve fascicular area over the sample length curve slopes were the closest to the 90 angle with the x axis in samples trimmed with a neurotome. The fascicular area of nerves trimmed with **E** scalpel and **F** scissors showed a gradual incline in fascicular area.

Evaluation of micro-CT image segmentation and fascicular area graphs showed fascicular radiographic consistency through the length of the nerve samples, except at the trimmed ends (Fig. 3A–C). The trimmed nerve ends of the neurotome group showed the steepest incline in fascicular area, consistent with a clean or equally dispersed incision (Fig. 3D). The trimmed ends of the scalpel and scissors groups showed a more gradual incline in fascicular area, indicating a more irregular cut compared to the neurotome (Fig. 3E, F).

**Figure 4:**
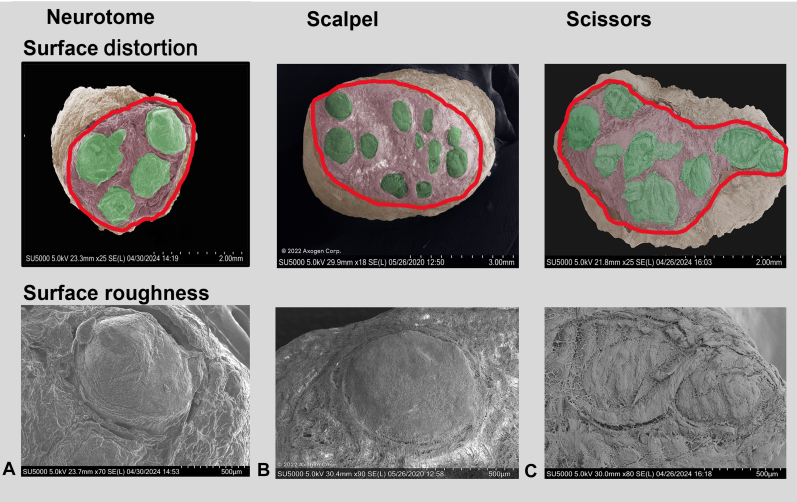
cSEM images. **A** Nerve end cut using a neurotome. **B** Nerve end cut using a scalpel blade. **C** Nerve end cut using scissors.

**Figure 5:**
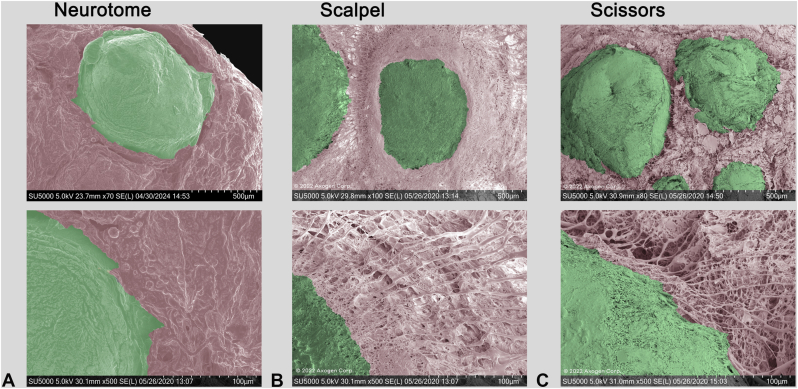
High magnification (top image is 100×, bottom image is 500×) cSEM images. **A** Nerve end cut using a neurotome. **B** Nerve end cut using a scalpel blade. **C** Nerve end cut using scissors. Surface roughness and collagen fiber separations were more notable in scissor-trimmed nerves.

The cut nerve end was visualized in cSEM images. Neurotome and scalpel cut nerve ends (Fig. 4A, B) qualitatively appeared to have less fascicular distortion and a smoother nerve face than nerve ends cut with scissors (Fig. 4C). Nerve surface morphological structure evaluation under high magnification cSEM showed no notable collagen separation between fascicules and internal epineurium in scalpel-trimmed and neurotome-trimmed nerves (Fig. 5A,B) and rough surface topography in scissor-trimmed nerves (Fig. 5C).

**Figure 6:**
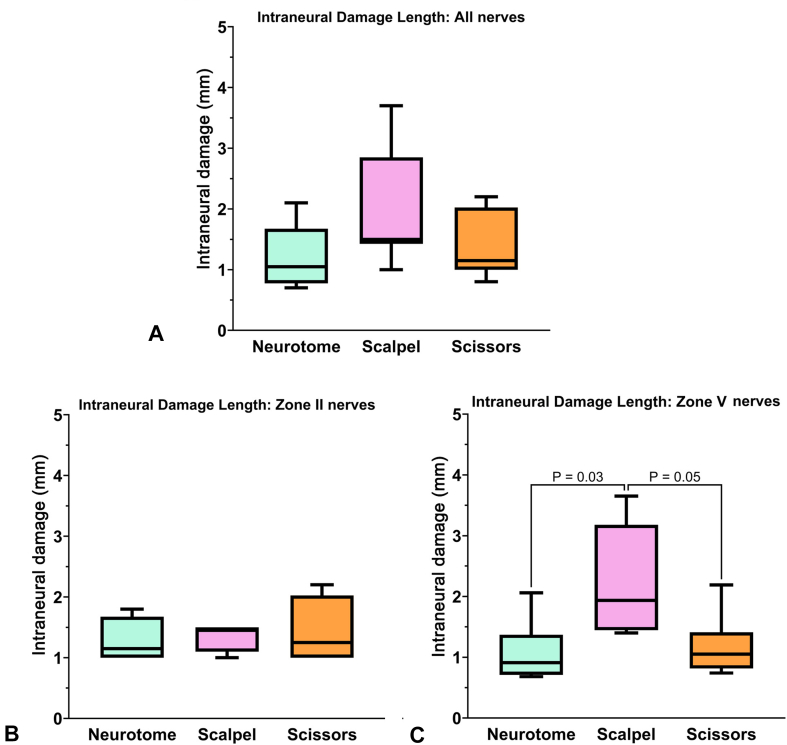
Radiographic intraneural damage length after nerve trimming with three different surgical instruments: **A** All nerves, showing no differences between groups (*P* = .08). **B** Zone II nerves, showing no significant differences between groups (*P* = .88). **C** Zone V nerves, showing significant differences between groups (*P* = .02). Neurotome and scissors groups showed significantly less intraneural damage length than the scalpel group (*P* = .03 and *P* = .05, respectively).

When all samples were pooled, there were no significant differences in radiographic analysis of trimmed nerve end intraneural damage across the three instruments (*P* = .08; Table 2, Fig. 6A). There were no significant differences in radiographic intraneural damage across the three instruments in zone II (*P* = .88, Fig. 6B). In zone V, there were significant differences across groups (*P* = .04). Additionally, there was significantly higher intraneural damage in the scalpel group compared to the neurotome group (*P* = .02, Fig. 6C) and borderline more intraneural damage in the scalpel group compared to the scissors group (*P* = .05). There were no significant differences between the neurotome group and the scissors group (*P* = .75).

**Table 2:** Surgical Instrument Effects on Nerve End Damage Comparison

Trimming Instrument	Sample size (n)	Mean (mm)	St Dev (mm)	Median (mm)	95% CI of Means (mm)	*P* Value
Data pooled for zone II and zone V						
Neurotome	8	1.21	0.50	1.05	0.79–1.63	.08
Scalpel	8	2.00	0.94	1.50	1.37–3.05	
Scissors	8	1.38	0.55	1.15	0.92–1.83	
Data for zone II						
Neurotome	4	1.28	0.38	1.15	0.67–1.88	.88
Scalpel	4	1.35	0.24	1.45	0.97–1.73	
Scissors	4	1.43	0.57	1.43	0.52–2.33	
Data for zone V						
Neurotome	4	1.15	0.66	0.90	0.10–2.20	.04
Scalpel	4	2.65	0.93	2.70	1.17–4.13	
Scissors	4	1.33	0.61	1.15	0.36–2.29	

CI, confidence interval; n, number of trimmed nerve ends used for micro-CT examination.

## Discussion

Optimal preparation of nerve stumps for repair or reconstruction should minimize internal nerve damage and should create a smooth nerve face. A common belief among surgeons is that the trimming of nerves influences patient outcomes after nerve transfers.^5^ Currently, various instruments are used in the surgical field to prepare nerve ends for repair or reconstruction; however, there is little to no consensus on the optimal surgical instrument for nerve trimming. Creating a flat surface that matches the surface of the opposing nerve face is generally accepted to optimize axonal regeneration after nerve coaptation.^5^ We hypothesized that fascicular integrity at the face of the trimmed nerve end and the length of intraneural damage would be different between cutting methods and instruments.

We recognize the inherent limitations of extrapolating morphologic results from cadaveric nerve tissue to clinical application; however, the highly magnified three-dimensional measurements provided by micro-CT analysis offer a more sensitive analysis of iatrogenic nerve surface damage from different cutting instruments than other evaluation methods in the literature.^1^^,^^3^, ^5^, ^4^^,^^7^^,^^11^ Published studies leveraging scanning electron microscopy found better fascicular and epineural integrity in nerve ends trimmed with razor blades compared to nerve ends trimmed with scalpel, a smoother nerve surface when cut with super-cut serrated microscissors versus nerve cutting guide forceps, and a smoother nerve surface when cut with no. 11 scalpel blade than cut with scissors and then trimmed with razor blades.^3^^,^^4^^,^^11^ Our study showed similarities, where cSEM evaluations showed that neurotome and scalpel-trimmed nerve end surfaces were smoother and had less distorted fascicles than scissor-trimmed nerve ends. These qualitative evaluations were notable; however, the micro-CT evaluations showed quantifiable differences between nerve cutting with neurotome and scalpel in zone V nerves, where the scalpel blade showed more extensive intraneural damage compared to the neurotome. These differences may be attributed to the nerve compression against the tongue depressor as the scalpel is guillotined through the nerve and displacement of the large diameter nerve fascicles, resulting in an uneven transection. Nerves evaluated by micro-CT showed similar intraneural damage across trimming instruments in small diameter nerves. We theorize that the small nerve diameter samples showed similarities across cutting samples because of the small surface area, as differences may have been more challenging to detect.

These results highlighted the neurotome-trimmed nerves as having optimal characteristics for subsequent peripheral nerve repair, as compared to scalpel or scissor. The comparison of these trimming instruments to other trimming instruments requires further investigation. Although there are many options for trimming nerves, we selected the instruments presented as they are common instruments in our experience and in literature. Our study identified that the neurotome-trimmed nerves consistently demonstrated less intraneural damage and less visible fascicular distortion. Our findings suggest that trimming the nerve face with a surgical blade, either neurotome or scalpel, was superior to the nerve face trimmed with super-cut serrated microscissors. Furthermore, we suggest that resecting injured nerves with a surgical blade may allow for optimal nerve repair and reconstruction outcomes.

## Conflicts of Interest

Fraser J. Leversedge, MD, Bauback Safa, MD, and Brandon S. Smetana, MD, are consultants for Axogen. Adam B Strohl, MD, is a consultant and on the advisory board for Axogen. Sunishka M. Wimalawansa, MD, is a consultant for Axogen and Checkpoint Surgical. Rasa Zhukauskas, MD, is an employee of Axogen. The other authors have no relevant conflicts to disclose.
